# Editorial for the Special Issue on Micro-Resonators: The Quest for Superior Performance

**DOI:** 10.3390/mi9120623

**Published:** 2018-11-27

**Authors:** Reza Abdolvand

**Affiliations:** Dynamic Microsystems Lab, Department of Electrical and Computer Engineering, University of Central Florida, Orlando, FL 32816, USA; reza@ece.ucf.edu

Micro-resonators have reached a distinctive level of maturity due to the accumulated wealth of knowledge on their design, modeling, and manufacturing during the past few decades [1]. Alongside this tremendous scientific progress, micro-resonators are now commonly found in most electronic systems. In this Special Issue, our attempt was to look deeper into less-common topics in this field, such as the nonlinear operation of micro-resonators that are envisioned to play a more important role with the evolution of this technological area.

As the energy density in a resonant device increases, the nonlinear effects could no longer be avoided or ignored. Therefore, it is critical to identify and carefully study the system parameters that impact nonlinearity in micro-resonators and to investigate the effect of nonlinearity in the performance. In Reference [2], the authors study how non-idealities, such as etch profile, in the fabrication of capacitive micro-resonators could affect nonlinear behavior of the device, and in Reference [3], a more accurate one-degree-of-freedom model is developed for the prediction of nonlinear behavior in capacitive beam resonators. Furthermore, in Reference [4], a novel resonator design is proposed to excite a 2:1 internal resonance through nonlinear coupling and to study the effect of air-damping loss on the operation of such devices.

Furthermore, the tuning range, frequency stability, and quality factor (Q) of micro-resonators are the focus in References [5,6,7] correspondingly, all of which are of significant practical importance, specifically in oscillator applications. The authors of [5] propose two methods for extending tuning range through stiffness alteration that could be effectively implemented in torsional resonators. In Reference [6], frequency stability in response to applied acceleration is investigated in bulk-extensional single crystalline silicon resonators and the dependency of acceleration-sensitivity on the resonator orientation with respect to the silicon crystalline planes are studied through finite element modeling and demonstrated through measurement. In Reference [7], the authors present the effectiveness of phononic crystal band-gap structures in improving the Q in bulk-extensional micro-resonators by reflecting acoustic energy back to the acoustic cavity, as they are strategically placed outside the anchors.

Finally, three unconventional micro-resonator structures are explored in References [8,9,10]. In Reference [8], the authors introduce a technique called chemical foaming to form glass bubbles that could be utilized for the implementation of hemispherical resonators. In Reference [9], an LC tank is presented with a significant size/performance enhancement achieved through the insertion of a coupling capacitance at the center of an air-bridged circular spiral inductor. Lastly, in Reference [10], the authors propose a unique approach to the realization of electromagnetically induced transparency (EIT) through cascaded multi-mode optical micro-ring resonators.

At the end of this brief introduction to the Special Issue, we would like to thank the authors who entrusted us with the publication of their scientific contributions and acknowledge the many expert reviewers whose technical insight has been instrumental in the timely evaluation of the submitted papers.
